# Birth asphyxia and its implications for neuropsychology and brain volume in schizophrenia

**DOI:** 10.1192/j.eurpsy.2021.186

**Published:** 2021-08-13

**Authors:** L. Wortinger, K. Jørgensen, C. Barth, I. Agartz

**Affiliations:** 1 Department For Psychiatric Research, Diakonhjemmet Hospital, Vinderen, Oslo, Norway; 2 Norment, Institute Of Clinical Medicine, University of Oslo, Oslo, Norway; 3 Department Of Clinical Neuroscience, Centre For Psychiatric Research, Karolinska Institute, Stockholm, Sweden

**Keywords:** Obstetric complications - Asphyxia, cognition, schizophrénia, Brain morphometry

## Abstract

**Introduction:**

Newborn infants can suffer permanent brain damage as a result of birth asphyxia (ASP), a severe obstetric complication (OC). However, effects of OCs on cognitive abilities and brain structure in schizophrenia (SZ) are unknown.

**Objectives:**

The main goals of this study were to investigate putative effects of a history of OCs on adult cognition and brain structure in SZ.

**Methods:**

We utilized prospective data from the Medical Birth Registry of Norway to identify incidences of severe OCs in adult healthy controls (HC; n = 622) and patients with SZ (n = 607). IQ was assessed, and a subset of participants (n= 414) underwent magnetic resonance imaging.

**Results:**

Severe OCs (27%) and ASP (14%) were equally common in SZ and HC. SZ patients with OCs had lower IQ than patients without OCs, a difference not found in HC (p = .023). Having experienced more than one co-occurring severe OC was associated with lower IQ in both groups, wherein 81% of co-occurring OCs involved ASP. ASP was related to smaller intracranial volume and brain volumes in both groups. Smaller caudate volumes were found in SZ patients with ASP compared to patients without ASP, a difference not found in HC (p = .009).

**Conclusions:**

Our findings give support for an effect of birth ASP on brain development in both patients with SZ and HC. OC history specifically impacts IQ in SZ. Smaller caudate volumes might be particularly related to disease development. These results warrant replication in an independent sample.

**Disclosure:**

No significant relationships.

